# The eEF1A Proteins: At the Crossroads of Oncogenesis, Apoptosis, and Viral Infections

**DOI:** 10.3389/fonc.2015.00075

**Published:** 2015-04-07

**Authors:** Wasim Abbas, Amit Kumar, Georges Herbein

**Affiliations:** ^1^Department of Biology, SBA School of Science and Engineering, Lahore University of Management Sciences, Lahore, Pakistan; ^2^UPRES EA 4266, Laboratory of Pathogens and Inflammation, Department of Virology, CHRU Besançon, Université de Franche-Comté, Besançon, France

**Keywords:** eEF1A, cancer, apoptosis, virus, HIV

## Abstract

Eukaryotic translation elongation factors 1 alpha, eEF1A1 and eEF1A2, are not only translation factors but also pleiotropic proteins that are highly expressed in human tumors, including breast cancer, ovarian cancer, and lung cancer. eEF1A1 modulates cytoskeleton, exhibits chaperone-like activity and also controls cell proliferation and cell death. In contrast, eEF1A2 protein favors oncogenesis as shown by the fact that overexpression of eEF1A2 leads to cellular transformation and gives rise to tumors in nude mice. The eEF1A2 protein stimulates the phospholipid signaling and activates the Akt-dependent cell migration and actin remodeling that ultimately favors tumorigenesis. In contrast, inactivation of eEF1A proteins leads to immunodeficiency, neural and muscular defects, and favors apoptosis. Finally, eEF1A proteins interact with several viral proteins resulting in enhanced viral replication, decreased apoptosis, and increased cellular transformation. This review summarizes the recent findings on eEF1A proteins indicating that eEF1A proteins play a critical role in numerous human diseases through enhancement of oncogenesis, blockade of apoptosis, and increased viral pathogenesis.

## Introduction

**Figure 1:**
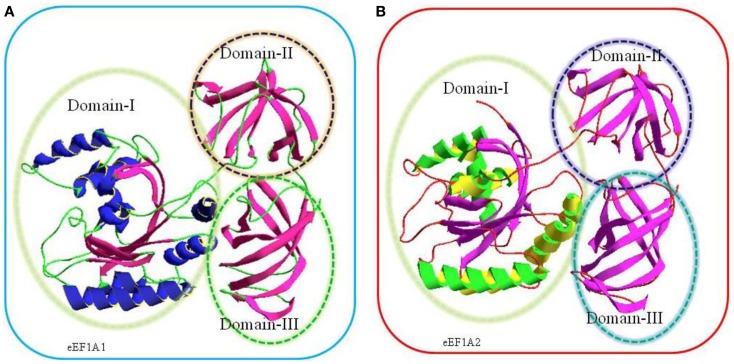
**Comparative three-dimensional (3-D) model of eEF1A1 (A) and eEF1A2 (B) on the basis of crystal structure of homologous eEF1A from yeast**. The target sequences used were eEF1A1 (Swiss-Prot Accession No: P68104) and eEF1A2 (Swiss-Prot Accession No: Q05639). The amino acids sequences of each protein were submitted to SWISS-MODEL server to build a 3D model (10).The highest resolved structure, 1.67-Å X-ray-derived eEF1A protein structure from yeast (*Saccharomyces cerevisiae*) with PDB ID: 1f60 and *E*-value 0.0 (sequence identity: 80.371%) was used as a template for modeling. Structurally, each model consists of three domains, domain I, domain II, and domain III as shown in the above cartoon. Domain I (residues 1–240) is made up of Rossmann-fold topology. Domain II (residues 241–336) and domain III (residues 337–443) are made up of entirely from beta-strands; each domain contains two beta sheets that form a beta barrel (7).

Translation is the central event leading to protein synthesis and translation factors are key actors involved in the translation process (1–3). Recently, it has been suggested that translation factors could represent a new class of potential oncoproteins that could favor cellular transformation through protein translation infidelity, association with cytoskeleton alterations, and modulation of signaling pathways (3–5). The eukaryotic translation elongation factors 1 alpha, eEF1A1 and eEF1A2, are the second most abundant protein (1–3% of total protein content) after actin and an important component of translation machinery. In its GTP bound form, eEF1A1 and eEF1A2 deliver the aminoacylated-tRNA to the A site of the ribosome for decoding of mRNA by codon–anticodon interactions (6). Following the hydrolysis of GTP, eEF1A (eEF1A1 and eEF1A2), GDP is released from the ribosome (6). Thus, eEF1A1 and eEF1A2 are GTP-binding proteins and consist of three domains namely domain I, domain II, and domain III. Domain I spans over 1–240 residues, which made up of a Rossmann-fold topology. Domain II (241–336 amino acids) and domain III (residues 337–443) consist of beta strands and each domain contains two beta sheets that form the beta barrel (7–10) (Figure 1).

There are two known isoforms of protein eEF1A, i.e., eEF1A1 and eEF1A2. The cellular expression of eEF1A is divided into three classes. First are the majority of cell types that express only eEF1A1. Second, neurons and muscle cells that express only eEF1A2. Third class belongs to certain tumor cell types and cell lines that express both eEF1A isoforms (11–13). eEF1A1 protein has been mapped on chromosomes 6q14. eEF1A1 protein shares homology with eEF1A2 protein at the nucleotide level (75%) and amino acid level (96%). eEF1A2 does not bind GDP and GTP with the same relative affinity as eEF1A1. The GDP dissociation rate constant is seven times higher for eEF1A1 than for eEF1A2. In addition, the nucleotide preference ratio (GDP/GTP) for eEF1A1 is 0.82 and for eEF1A2 is 1.50 (14–17). Furthermore, eEF1A has been shown to be a novel component of the nuclear export machinery in mammalian cells and is involved in the nuclear export of specific proteins such as VHL van Hippel-Lindau (VHL) tumor suppressor and poly(A)-binding protein (PABP1) (18).

**Figure 2:**
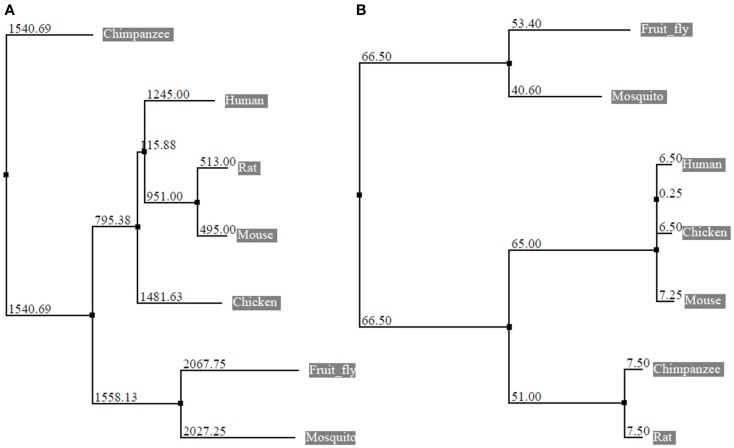
**Phylogenetic tree constructed from the alignment of nucleotide and protein sequences of eEF1A2**. The horizontal lines are the branches and suggest the amount of evolutionary changes over time. **(A)** Phylogenetic tree based on nucleotide sequences of eEF1A2 (Human: NM_001958.3, Chimpanzee: XM_003954094.1, Rat: NM_012660.2, Mouse: NM_007906.2, Fruit fly: NM_079872.4, Chicken: NM_001032398.2, and Mosquito: XM_003436467.1) using neighbor-joining distance method. The numbers indicate the evolutionary distances. **(B)** Phylogenetic tree based on amino acid sequences of eEF1A2 (Human: NP_001949.1, Chimpanzee: XP_003954143.1, Rat: NP_036792.2, Mouse: NP_031932.1, Fruit fly: NP_524611.1, Chicken: NP_001027570.1, and Mosquito: XP_003436515.1 using neighbor-joining (PAM250). The Jalview program was used for tree construction.

**Figure 3:**
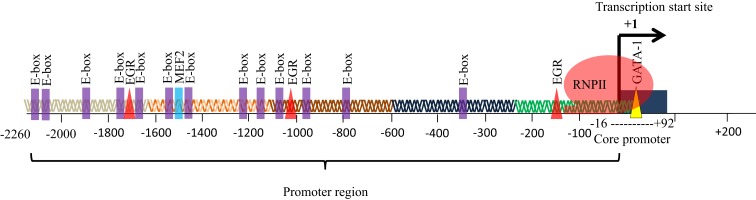
**Promoter of eEF1A2**. The promoter of eEF1A2 spans approximately 2.26 kb. Analysis of promoter sequence reveals 13 E-boxes, 3 EGR binding sites, and 1 MEF2 binding site. The core promoter region spans from position −16 to +92 bp.

The eEF1A2 gene is present in the common ancestor of eukaryotes (Table 1; Figure 2). Human eEF1A2 gene spans approximately 12 kb human genome sequence, which consists of eight exons and seven introns plus 2 kb upstream promoter region, and has been mapped on chromosome 20q13.3. Analysis of the region −2064 to +220 reveals that the promoter region contains the binding sites for several important cis-regulatory elements (E-boxes, EGR family proteins, GATA motif, and MEF binding site) with no TATA elements (Figure 3). The core region of the promoter is mapped from position −16 to +92 (9, 19). Beyond its central role in translation machinery, eEF1A2 plays an important role in cell cycle regulation, heat-shock response, aging, posttranslational modifications, and signal transduction (20–23).

**Table 1:** **Orthologs for eEF1A2 gene**.

Organism	Similarity with human	
	Nucleotide (%)	Amino acid (%)
Chimpanzee	78.31	92.41
Rat	89.27	99.78
Mouse	89.13	99.78
Chicken	88.91	99.57
Mosquito	78.99	85.42
Fruit fly	77.68	83.91

Protein synthesis is one of the most sophisticated biochemical processes occurring in the cell, which requires hundred of proteins, including eEF1A proteins, eEF1A1 and eEF1A2. Besides the role of eEF1A proteins in the translational process, an increasing series of data are presently emerging about the non-canonical roles of these proteins in oncogenesis, modulation of apoptosis and viral pathogenesis (1, 20).

## Cancer

### Breast cancer

**Figure 4:**
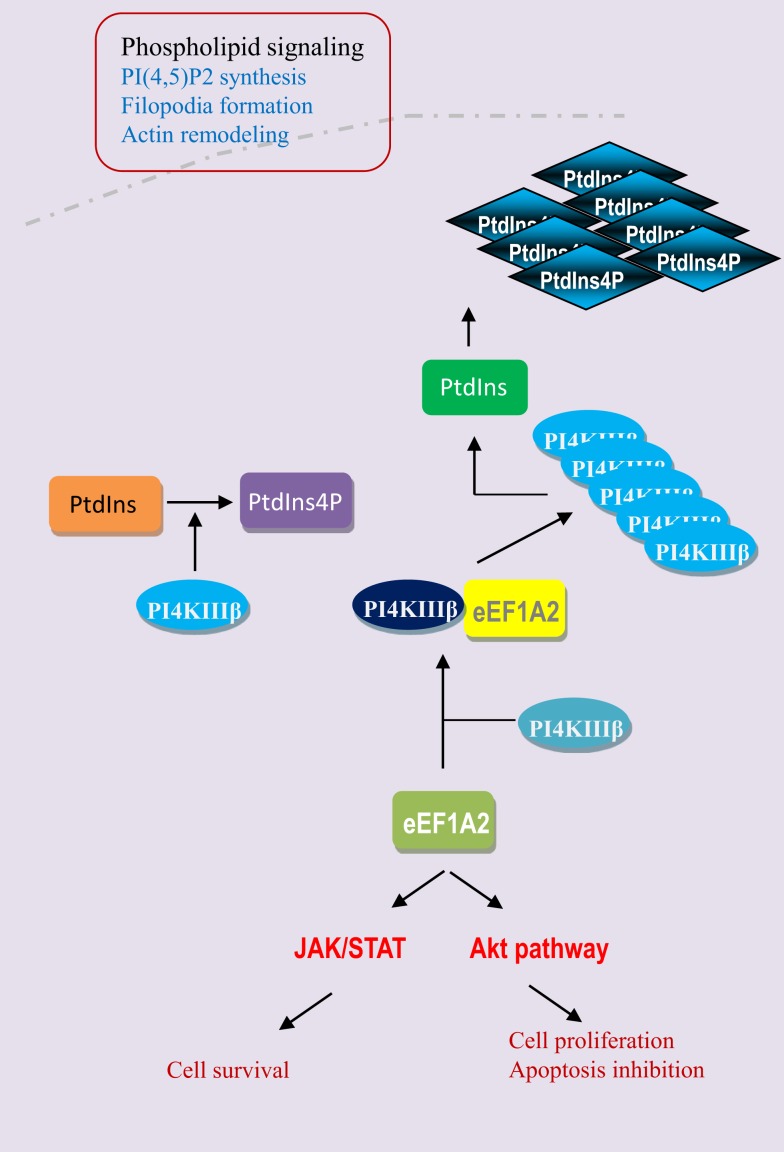
**eEF1A2 activates the phospholipid, JAK/STAT, and Akt pathways**. eEF1A2 is a physiological regulator of phosphatidylinositol-4-kinase (PI4K). It directly interacts with phosphatidyloinositol-4 kinase III β (PI4KIIIβ) and enhances its lipid kinase activity by converting the phosphatidylinositol (PtdIns) into phosphatidylinositol-4-phosphate (PtdIns4P). Then PI4KIIIβ and PtdIns4P increase the phosphatidylinositol-4,5 bisphosphate generation at the plasma membrane level, which results in the filopodia formation and actin remodeling. eEF1A2 also directly or indirectly regulates the JAK/STAT and Akt signaling pathways.

Breast cancer is the most common cancer in women both in the developing and developed world; there are an estimated 1 million new cases per year. The use of gene signature or the identification of changes in gene expression in breast tumors relative to normal surrounding tissue is of great importance in terms of prognostic indicators and therapeutic targets (24). The eEF1A2 is hardly detectable in normal human breast tissue but the expression of eEF1A2 is strongly upregulated in most of breast tumors (25, 26). High levels of eEF1A2 proteins are detected in 60% of primary breast tumors and metastases, but not in normal epithelium (Table 2). The expression of eEF1A2 is sufficient to stimulate the formation of filopodia in BT549 human breast cancer cells and non-transformed Rat2 cells. Moreover, its expression is sufficient to activate Akt in a PI3K-dependent fashion, as down-regulation of eEF1A2 by siRNA reduces Akt activity. In breast cancer cell line BT549, eEF1A2 expression stimulates cell migration and invasion in a largely PI3K and Akt-dependent manner, suggesting eEF1A2 regulates oncogenesis through Akt and PI3K-dependent cytoskeleton remodeling (27–29). In fact, eEF1A2 participates in the regulation of the phospholipids signaling pathway (Figure 4). Phosphatidylinositols are negatively charged membrane bound phospholipids that participate in the pathways that regulate the cell proliferation, survival, cytoskeleton organization, vesicular trafficking, and oncogenesis (30, 31). Phosphoinositols are composed of an inositol ring in which one or more −OH groups at the 3-, 4-, and 5-position of inositol ring are esterified with a phosphate group in all possible combinations. Specific kinase families (PI3K, PI4K, and PI5K) are responsible for phosphorylation at these sites (32, 33). Overexpression of eEF1A2 protein up-regulates overall PI4K activity and cellular phosphatidylinositol 4-phosphate (PI4P) generation in human cells. Furthermore, eEF1A2 directly interacts with and activates phosphatidylinositol-4 kinase III β (a subfamily of PI4K), an enzyme that converts phosphatidylinositol to PI4P. Knockdown of eEF1A2 using eEF1A2 siRNA results in down-regulation of phosphatidylinositol-4-kinase activity indicating that eEF1A2 is a physiological regulator of PI4KIIIβ signaling (34, 35). In addition, eEF1A2 expression up-regulates the generation of phosphatidylinositol-4,5-bisphosphate [PI(4,5)P2] in the cytoplasm and at the plasma membrane. The subsequent increase in PI(4,5)P2 at the plasma membrane stimulates the eEF1A2-induced filopodia formation through binding and activation of PI4KIII β. Therefore, eEF1A2 is involved in phosphatidylinositol signaling and actin remodeling (34, 36). Moreover, the gene expression profiling of primary mouse B cell lineage showed the high expression of eEF1A2 in plasmacytomas (PCT), which results in progression of plasma cell neoplasms in both mouse and human (29). Finally, the knockdown of eEF1A2 expression delays or impairs the IL-6-induced activation of STAT3 and Akt signaling pathways suggesting that activation of STAT3 and Akt through eEF1A2 involvement that favors cell proliferation, cell cycle progression, and inhibition of apoptosis (29, 37, 38). Altogether, eEF1A2 protein activates the PIK-Akt-STAT3 pathways that have been extensively shown to favor cellular transformation and oncogenesis (39–44) (Figure 4).

**Table 2:** **Expression of eEF1A2 in different human cancers**.

Type of cancer	Methods of detection	Relevant findings	Reference
Breast cancer	Real-time reverse transcription-PCR/Tissue array/Immunohistochemistry/western blot	eEF1A2 mRNA/protein is highly expressed in 50–60% in primary human breast cancer	(25, 39)
Ovarian cancer	Tissue-microarray/Immunohistochemistry	eEF1A2 is highly expressed in 30% of primary ovarian tumors	(40, 56)
Lung cancer	Comparative genomic hybridization	Positive Ki-67 expression associated with positive eEF1A2 and KCIP-1	(41, 42)
Hepatocellular carcinoma	Immunohistochemistry	eEF1A2 is highly expressed in half of hepatocellular carcinoma	(43, 44)

In breast cancer, the genes in 20q13 are frequently amplified and have been shown by using comparative genomic hybridization and fluorescence *in situ* hybridization (45). The differential screening of cDNA libraries from metastatic and non-metastatic cell lines derived from the same parental rat mammary adenocarcinoma showed a 1.5-fold overexpression of eEF1A in the metastatic compared to the non-metastatic cells (46). Studying cancer cell lines derived from breast, lung, prostate, and skin, eEF1A2 gene expression exhibited the highest alteration in the cancer cell lines compared to normal controls using a profiling array (47–50).

### Ovarian cancer

Ovarian cancer represents 4% of all female cancer. It has the highest fatality to case ratio of all gynecological cancers because the majority of cases are diagnosed in the late stage. Despite of significant efforts to improve the early detection and advances in chemotherapy, metastasis remains a major challenge in clinical management of ovarian cancer (51–54). The eEF1A2 gene is not expressed in normal ovary but highly expressed in ovarian cancer (55). eEF1A2 expression enhances the ovarian cell growth *in vitro* and favors the tumorsphere formation (56), suggesting that eEF1A2 could favor the development of ovarian primary tumor formation. High levels of eEF1A2 expression was observed in 30% all the primary ovarian tumors, 50% of serous tumors, 30% of endometrioid tumors, 19% of mucinous tumors, and 8% of clear-cell tumors (Table 2) (56). Furthermore, the eEF1A2 protein and RNA expression levels are upregulated in clear-cell ovarian carcinoma by 75 and 33% respectively. The eEF1A2 gene is unmethylated both in normal and tumor cells, suggesting that up-regulation of eEF1A2 gene expression is not dependent on epigenetic modifications (at least for the methylation status), but instead that the inappropriate expression of trans-acting factor(s) could be involved (55). The enhanced expression of eEF1A2 protein in ovarian cancers correlates with poor prognosis (57).

The oncogenic properties of eEF1A2 have also been studied in different ovarian tumors and established cell lines. In rodent fibroblasts, eEF1A2 protein favors anchorage-independent growth and results in decreased doubling time during cellular proliferation. Furthermore, the induced expression of eEF1A2 in NIH3T3 cells makes them tumorigenic and increases the growth rate of ES-2 ovarian carcinoma cells xenografted in nude mice (40). The transcription factor ZNF217 and eEF1A2, both located on chromosome 20q13, are frequently amplified in ovarian epithelial carcinomas. The stable preneoplastic ovarian cell lines that over-express eEF1A2 provides the evidence that up-regulation of eEF1A2 expression contributes to the neoplastic properties of precursor cells of ovarian carcinomas mediated through ZNF217 (58).

Resveratrol, a phytoalexin produced naturally by several fruit plants, has been extensively studied for its chemopreventive and chemotherapeutic effects in cancer and animal models. Resveratrol blocks the angiogenesis, induces the autophagocytosis and apoptosis in proliferating cells, and is a well-known sirtuin 1 activator (59, 60). The expression of eEF1A2 is increased in the PA-1 ovarian cell line after serum or insulin stimulation. Resveratrol up-regulates the caspase-3 level in PA-1 cells by down regulating the expression of eEF1A2 via the blockade of upstream Akt pathway. Additionally, resveratrol suppresses growth of human ovarian cancer cells in culture and in a murine xenograft model with reduced expression of proliferating cell nuclear antigen and increased TUNEL staining (61). All together resveratrol down-regulates eEF1A2 in ovarian cancer cells and thereby favors apoptosis.

### Lung cancer

Lung cancer is the most common cause of cancer related death both in men and women. It accounts for 1.3 million deaths worldwide annually. In spite of continuous efforts and clinical trials to develop new diagnostic and prognostic markers, a better understanding of the molecular pathways involved in lung cancer is essential for developing new therapeutic targets (62, 63). Four lung adenocarcinoma cell lines (HKULC 1–4) were established from patients with different clinical characteristics. Comprehensive studies of these cell lines show that eEF1A2 is a putative oncogene that is highly expressed in all the HKULC cell lines (64). In addition, high-resolution analysis of genomic aberration by metaphase and comparative genomic hybridization array identify the involvement of the 20q region, suggesting the potential role of eEF1A2 as a candidate tumor gene in lung cancer cell lines (41). In another study, 183 genes with increment in both genomic copy number and transcript in six lung adenocarcinoma were analyzed. Forty-two proteins were overexpressed in these cell lines as compared to the normal cells. Comparing the 183 genes with the 42 proteins, the expression of four candidates, namely, PRDX1, eEF1A2, CALR, and KCIP-1 was correlated with increased DNA copy number and transcript levels. Furthermore, expression of siRNA targeting eEF1A2 and KCIP-1 in these cell lines suppressed cellular proliferation and triggered apoptosis. Therefore, the overexpression of eEF1A2 and KCIP-1 in lung tumor samples strongly suggests that both proteins could be involved in lung adenocarcinoma and could be potential therapeutic targets in lung cancer (42).

### Liver cancer

Hepatocellular carcinoma (HCC) is one of the most common malignant tumors in humans (65, 66). HCC cell lines, HepG2, HuH7, and JHH6 show increased expression of eEF1A2 as compare to normal liver tissue, but the mRNA of eEF1A2 is markedly increased only in JHH6 cells (43). In addition, the MDM4 and eEF1A2 proteins show increased expression in cryptogenic HCC (44). eEF1A2 gene silencing reduces cell viability, proliferation, and increases the apoptosis rates in HCC cell lines (44). Furthermore, eEF1A2 is overexpressed in 43% of HCC and activates the Akt pathway as observed in 40–60% of primary HCCs (Table 2) (44, 67). Using array based comparative genomic hybridization, frequent amplification and gain of DNA copy number at 1q, 6p, 8q, 17q, and 20q were observed in HCCs, suggesting that these genetic aberrations might facilitate the malignant transformation (68).

### Pancreatic cancer

Pancreatic cancer is one of the unsolved health problems associated with aggressive malignancies and therefore there is need to develop improved early diagnosis tools (69, 70). The expression of eEF1A2 gene, located on chromosome 20q13 is significantly upregulated in pancreatic cancer. Although little or no eEF1A2 expression is present in normal pancreatic tissue, 83% of pancreatic cancers display increased expression of eEF1A2, suggesting that eEF1A2 plays an important role in pancreatic carcinogenesis (71–73).

## Apoptosis

Cell proliferation and cell death are highly regulated and coordinated during the normal development, and both are crucial for the maintenance of tissue homeostasis. Impaired apoptosis can lead to autoimmunity or malignancy (74, 75). An increasing number of evidence suggests the involvement of eEF1A2 at the onset of cell transformation. Peroxiredoxin-1 (Prx-1) is a protein that is ubiquitously expressed in all mammalian cells and protects the cells from oxidative stress by reducing the range of reactive oxygen species. eEF1A2 interacts directly with Prx-1 and protects the cells from stress-induced apoptosis by the down-regulation of caspase-3 and caspase-8 activation parallel to increased expression of the pro-survival factor Akt (76, 77). Additionally, the eEF1A expression varies in cells treated with various concentrations of hydrogen peroxide, a strong apoptotic inducer associated with decreased mitochondrial respiration and an inhibitor of Prx-1 (78, 79). Finally, eEF1A provides protection against all endoplasmic reticulum (ER) stress-mediated cell death. FL5.12 cells are non-transformed murine B-cells that require IL-3 for growth, survival, and proliferation. Enforced expression of eEF1A in these cells protects them from IL-3 withdrawal without cellular transformation (80).

Wasted mouse is a spontaneous autosomal recessive mutation associated with neurological defects, immune system abnormalities, and defective response to DNA damage repair in immune cells. Thymus and spleen from wasted mice show extensive apoptosis. The loss of activity in wasted mice and immunological abnormalities are solely linked to the presence of a deletion in the *eef1a2* gene (81–84). Furthermore, eEF1A2 is highly expressed in terminally differentiated cells such as neurons, cardiomyocytes, and myofibers. During skeletal muscle development, undifferentiated myoblasts are susceptible to serum deprived caspase 3-mediated apoptosis but become resistant to apoptosis after differentiating into myotubes. In muscle cells, the eEF1A1 is expressed during embryonic development of myoblast but is replaced by eEF1A2 after 2 weeks of birth indicating that the eEF1A1 isoform plays a pro-apoptotic role while the eEF1A2 isoform displays anti-apoptotic properties (85, 86).

## Viral Infections

**Figure 5:**
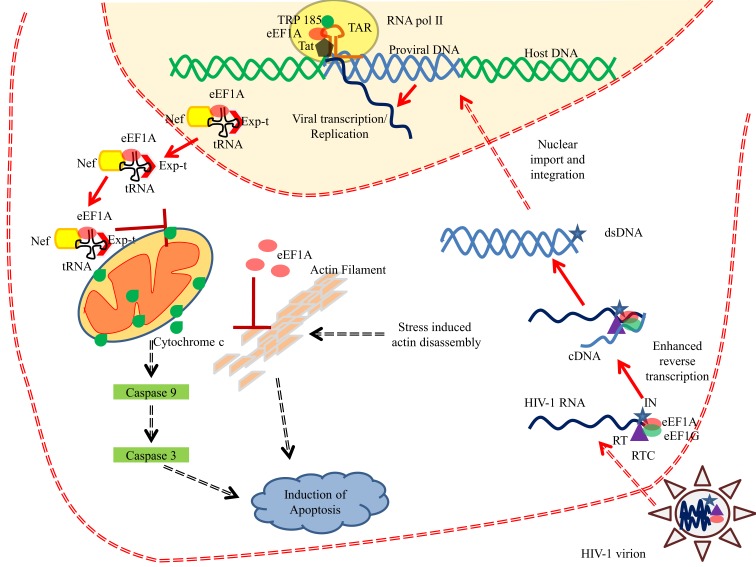
**eEF1A interferes with HIV-1 replication**. eEF1A plays important role in various phases of HIV-1 life cycle. eEF1A has been reported in the mature HIV-1 virion and is also found to be a part of reverse transcription complex (RTC). Binding of eEF1A/EF1G to the RTC resulted in enhanced reverse transcription. In addition, eEF1A also help in the recruitment of RNA polymerase II and TRP-185 to the TAR RNA, which in turn regulates the viral transcription from 5′LTR. Moreover, HIV-1 Nef has been shown to interact with eEF1A and resulted in nucleo-cytoplasmic shuttling of eEF1A and ultimately in the inhibition of stress-induced apoptosis. Role of eEF1A in inhibiting the actin filament disassembly has been also proposed (77). Abbreviations: eEF1A, eukaryotic translation elongation factor 1 alpha; Exp-t, exportin-t; dsDNA, double stranded DNA; cDNA, complementary DNA; IN, integrase; RT, reverse transcriptase; RTC, reverse transcription complex; tRNA, transfer RNA; TAR, trans-activation response element; RNA pol II, RNA polymerase II; Tat, transactivator protein.

The eEF1A proteins play a critical role in several viral infections at distinct stages of the viral cycle [reviewed in Ref. (87)] (Table 3). Among the most studied viruses, human immunodeficiency virus (HIV) interacts with numerous cellular proteins, including eEF1A (88). First, eEF1A has been detected as part of the HIV virions as an actin-binding protein (89). Second, eEF1A has been reported to plays a critical role at early stages of HIV replication (Figure 5). eEF1A and also EF1G are part of HIV reverse transcription complex (RTC) (90). Both eEF1A and EF1G proteins coimmunoprecipitated with the p51 subunit of reverse transcriptase (RT) and integrase using an endogenous reverse transcription assay (90) (Figure 5). The depletion of eEF1A and EF1G using siRNAs decreased reverse transcription parallel to reduced levels of RTC in treated cells (90). Since integrase is also an RT binding protein, a tight interplay between RT, integrase, and eEF1A could be involved in several stages of HIV replication. Actually, integrase has been shown to interact with eEF1A *in vitro* (91, 92). eEF1A also interacts with other viral proteins involved in early stages of HIV replication such as Nef and Tat. Several reports indicate that HIV Nef interacts with eEF1A (77, 93) (Figure 5). Abbas and colleagues have shown that the Nef-eEF1A interaction favors the nucleo-cytoplasmic shuttling of eEF1A and ultimately inhibits stress-mediated apoptosis in monocyte-derived macrophages (77). Additionally, Nef interaction with actin impairs human podocyte actin cytoskeletal integrity and eEF1A could play a role in the development of podocyte phenotype in HIV-1 associated nephropathy (93). The trans-activation response (TAR) element is critical for the activation of HIV-1 transcription. eEF1A stimulates the RNA polymerase II and TRP-185 binding to TAR RNA, which in turn regulates the HIV-1 gene expression (94). eEF1A has been described to interact with the HIV Gag protein (88). Finally, eEF1A is responsible for the selection and binding of cognate aminoacyl-tRNAs to the A site of the ribosome. HIV Nef interacts with two components of the 40S small ribosomal subunit, the RPS10 protein, and the 18S rRNA, and also to tRNAs, and has been reported to decrease translation using an *in vitro* translation assay (95). We cannot exclude that eEF1A that binds to both Nef and tRNA participates in the control of translation in HIV-infected cells. Interestingly, EF1-delta has been reported to interact with the second coding exon of HIV Tat, and to result in reduced efficiency of the translation of cellular proteins, but not of viral mRNAs (96).

**Table 3:** **Virus-eEF1A interplay**.

Virus	Description of eEF1A/viral protein interaction	Reference
HIV	eEF1A part of HIV-1 virion and reverse transcriptase complex	(77, 89, 90, 93)
	eEF1A also interacts with Nef and modulate apoptosis in MDMs	
HBV	HBx interacts with eEF1A1 resulting in blockade of actin bundling	(97)
HDV	HDV genome also interacts with eEF1A1	(98)
HPV 38	E7 protein interacts with both eEF1A1 and eEF1A2. The interaction is associated with cellular immortalization and transformation of primary keratinocytes	(99)
WNV	eEF1A interacts with the 3′stem–loop region of the viral genomic RNA and favors replication	(100, 101)
DENV	Viral genomic RNA sequesters eEF1A and hence decreases the concentration of Sphk1, thereby governing cell survival	(102)
TGEV	eEF1A interacts with the nucleocapsid of the virus and favors virus replication	(103)
VSV	eEF1A is found within the virion	(104)
Vaccinia virus	eEF1A as a part of mature virion	(105, 106)
CMV	eEF1A found to be as a part of virion proteome	(107, 108)
SARS-CoV	eEF1A found in mature virion	(109)

*HIV, human immunodeficiency virus; MDMs, monocyte-derived macrophages; HBV, hepatitis B virus; HDV, hepatitis delta virus; HPV, human papillomavirus; WNV, West Nile virus; DENV, dengue virus; VSV, vesicular stomatitis virus; CMV, cytomegalovirus; TGEV, transmissible gastroenteritis coronavirus; SARS, severe acute respiratory syndrome-associated coronavirus (SARS-CoV)*.

Since overexpression of eEF1A has been reported in several cancers, we cannot exclude a link between oncoviruses and eEF1A. Among oncoviruses, hepatitis B virus expresses the HBx protein that has been previously involved in liver cancer, especially HCC (110). HBx protein has been reported to interact with eEF1A1 in Huh-7 hepatoma cells infected with recombinant adenovirus expressing HBx protein (97). Interaction of HBx protein with eEF1A1 blocks filamentous actin bundling (97). Interestingly, the hepatitis delta virus (HDV) that can propagate only in the presence of HBV has a RNA genome that also interacts with eEF1A1 (98). A dozen of human papillomavirus (HPV) types including HPV16 and HPV18 are well-known oncoviruses that express two oncoproteins E6 and E7, and favor cellular transformation, oncogenesis, and the appearance of cervical cancers (111). E7 protein of HPV38 has been shown to interact with both eEF1A1 and eEF1A2 proteins leading to cellular immortalization and transformation of primary keratinocytes probably through disruption of actin stress fiber formation, a critical event linked to tumor formation (99). eEF1A facilitates virus replication complex (RPC) assembly and favors replication of West Nile virus and dengue virus (100–102). Similarly, eEF1A interacts with the nucleocapsid protein of transmissible gastroenteritis coronavirus (TGEV) and favors TGEV replication (103). Since eEF1A is found in highly purified virions of numerous RNA and DNA viruses including vesicular stomatitis virus (104), vaccinia virus (105, 106), cytomegalovirus (107, 108), severe acute respiratory syndrome coronavirus (SARS-CoV) (109), and HIV-1 (88, 112), its role in viral pathogenesis needs to be further investigated.

## Conclusion

Recent findings in the field of cellular and molecular biology reveal that the eEF1A proteins are not only involved in the translational process but display also non-canonical functions. The specific up-regulation of eEF1A2 in numerous tumors and its transforming properties indicate that it could play a significant role in tumorigenesis. Furthermore, eEF1A2 activates the phospholipid and Akt signaling pathways favoring cell survival. Additionally, eEF1A proteins block apoptosis and favors viral replication. All together, the non-canonical functions of eEF1A proteins are involved at the crossroads of oncogenesis, blockade of apoptosis, and viral pathogenesis. Therefore, the eEF1A proteins might play an important role in the pathophysiology of tumors and apoptosis, especially in response to stress and viral infections. Future studies need to be done to further highlight the role of eEF1A proteins in human diseases.

## Author Contributions

WA and AK were responsible for drafting and revising the manuscript. GH was involved in critical reading of the manuscript.

## Conflict of Interest Statement

The authors declare that the research was conducted in the absence of any commercial or financial relationships that could be construed as a potential conflict of interest.
